# Prophylactic onlay mesh placement techniques for optimal abdominal wall closure: randomized controlled trial in an *ex vivo* biomechanical model

**DOI:** 10.1093/bjs/znad062

**Published:** 2023-03-15

**Authors:** Ian Stephens, Jack Conroy, Des Winter, Ciaran Simms, Magda Bucholc, Michael Sugrue

**Affiliations:** Department of Surgery, Letterkenny University Hospital, Letterkenny, Ireland; Donegal Clinical Research Academy, Letterkenny University Hospital, Letterkenny, Ireland; Trinity Centre for Bioengineering, Department of Mechanical, Manufacturing and Biomedical Engineering, Trinity College Dublin, Dublin, Ireland; Department of Surgery, St Vincent’s University Hospital, Dublin, Ireland; Trinity Centre for Bioengineering, Department of Mechanical, Manufacturing and Biomedical Engineering, Trinity College Dublin, Dublin, Ireland; EU INTERREG Centre for Personalized Medicine, Intelligent Systems Research Centre, School of Computing, Engineering and Intelligent Systems, Ulster University, Derry-Londonderry, UK; Department of Surgery, Letterkenny University Hospital, Letterkenny, Ireland; Donegal Clinical Research Academy, Letterkenny University Hospital, Letterkenny, Ireland

## Abstract

**Background:**

Incisional hernias occur after up to 40 per cent of laparotomies. Recent RCTs have demonstrated the role of prophylactic mesh placement in reducing the risk of developing an incisional hernia. An onlay approach is relatively straightforward; however, a variety of techniques have been described for mesh fixation. The biomechanical properties have not been interrogated extensively to date.

**Methods:**

This *ex vivo* randomized controlled trial using porcine abdominal wall investigated the biomechanical properties of three techniques for prophylactic onlay mesh placement at laparotomy closure. A classical onlay, anchoring onlay, and novel bifid onlay approach were compared with small-bite primary closure. A biomechanical abdominal wall model and ball burst test were used to assess transverse stretch, bursting force, and loading characteristics.

**Results:**

Mesh placement took an additional 7–15 min compared with standard primary closure. All techniques performed similarly, with no clearly superior approach. The minimum burst force was 493 N, and the maximum 1053 N. The classical approach had the highest mean burst force (mean(s.d.) 853(152) N). Failure patterns fell into either suture-line or tissue failures. Classical and anchoring techniques provided a second line of defence in the event of primary suture failure, whereas the bifid method demonstrated a more compliant loading curve. All mesh approaches held up at extreme quasistatic loads.

**Conclusion:**

Subtle differences in biomechanical properties highlight the strengths of each closure type and suggest possible uses. The failure mechanisms seen here support the known hypotheses for early fascial dehiscence. The influence of dynamic loading needs to be investigated further in future studies.

## Teaser

This *ex vivo* randomized controlled trial using porcine abdominal wall investigated the biomechanical properties of three techniques for prophylactic onlay mesh placement at laparotomy closure and compared them with small-bite primary closure. A biomechanical abdominal wall model and ball burst test were used. All techniques held up to extreme quasistatic loads, each with its own unique biomechanical profile.

## Introduction

Effective abdominal wall closure is an essential part of general surgical practice. Despite rapid advances in minimally invasive techniques, open access will continue to remain relevant in technically challenging and emergency procedures. Midline laparotomy incisions carry significant long-term morbidity, with incisional hernia being the most frequent complication^1,2^. Over 4 million laparotomies are performed annually in the USA^3^, with subsequent incisional hernia rates ranging from 20 per cent in unselected patient populations to almost 40 per cent in high-risk cohorts^4,5^. Incisional hernias are symptomatic in up to 84 per cent of patients^3^, with a 5-year revision rate of over 20 per cent^6^, and many present as an emergency with either incarceration (6–15 per cent) or strangulation (2 per cent)^7,8^.

Prophylactic mesh placement at the time of abdominal closure has been shown to reduce incisional hernia rates from 20–40 to 3.9–16 per cent^9–12^. However, its use may be associated with a trend towards increased chronic pain (7.8 per cent) and seroma formation (12.9 *versus* 6.9 per cent)^11^. Synthetic meshes have been shown to be safe for use even in the setting of peritonitis^10^, and long-acting resorbable biosynthetic meshes, such as TIGR^®^ matrix mesh (Novus Scientific AB, Uppsala, Sweden), have been used safely for abdominal closure^13^ without significant infection or seroma, including in a cohort of high-risk patients undergoing emergency laparotomy^14^. A recent multicentre RCT^15^ published in the USA demonstrated significantly lower rates of incisional hernia at 2 years, and median mesh cost, with use of a synthetic mesh (5.6 per cent; US $105/€99) compared with a biological mesh (20.5 per cent; $21 539/€20,280), with no difference in the rates of surgical-site occurrence.

Onlay mesh placement is a straightforward technique compared with complex abdominal wall reconstruction approaches, such as component separation. A classical onlay technique involves fixing the mesh to the anterior rectus fascia with an overlap of over 3 cm after primary closure of the linea alba^12^. The issue with the classical onlay method is that it does not directly buttress the fascial edge closure. It remains to be seen whether incorporating the mesh into the closing fascial suture would offer a biomechanical advantage.

This study used post-mortem porcine abdominal wall specimens as an *ex vivo* model to compare the biomechanical properties of standard suture primary closure, with those of a classical onlay technique, an augmented anchoring onlay method, and a novel bifid mesh-incorporating closure.

## Methods

### Sample preparation, randomization, and closure techniques

Porcine abdominal walls were collected fresh from an abattoir and frozen on arrival at the laboratory. Owing to practical constraints of storage, transport, and funding, testing was limited to 20 abdominal walls. Samples were removed from the freezer and thawed for a minimum of 24 h before testing. The samples were prepared by removing skin and subcutaneous fat, leaving just the abdominal fascia, musculature, preperitoneal fat, and peritoneum. For all but the initial four samples, this preparation was undertaken before freezing to reduce the need for prolonged thawing times associated with the larger unprepared samples.

Simple randomization was used. Twenty computer-generated numbers were randomly assigned to one the four closure methods—primary closure, classical onlay, anchoring onlay, or bifid onlay—in a 1 : 1 : 1 : 1 allocation ratio. After the abdominal incision had been made, a number was drawn blindly by the surgeon. A second investigator checked the number against its assigned closure method. This method was then used for the abdominal closure. This process was repeated for each consecutive abdominal wall across 4 testing days until no samples remained. Two samples were discarded owing to putrefaction before closure allocation. This left a total of 18 abdominal walls for allocation and testing.

A 10-cm supraumbilical incision was made in the midline of each sample, through the linea alba using a template. A 2/0 polypropylene suture (Prolene^®^; Ethicon, Somerville, NJ, USA) was used for all suturing. A small-bite technique (5 mm separate, 5 mm width) was used for primary midline closure. A 15 × 6-cm rectangular TIGR^®^ matrix mesh was used for all mesh closures.

In the classical closure group, primary closure was augmented by placement of mesh over the anterior fascia, with a 3-cm overlap on either side of the incision. The mesh was fixed in place with a running, locking suture placed circumferentially around its outer borders, tacking the mesh to the anterior fascia.

In the anchoring group, three interrupted sutures were placed through the full thickness of the linea alba and left untied before primary closure. These were placed at the apices and in the middle of the incision. Double-ended needles were left on these sutures, which were passed through the mesh after primary closure. The sutures were tied and cut, providing three points of fixation between the mesh and the full-thickness abdominal incision. The mesh was then fixed circumferentially in the same manner as used in the classical approach.

**Fig. 1:**
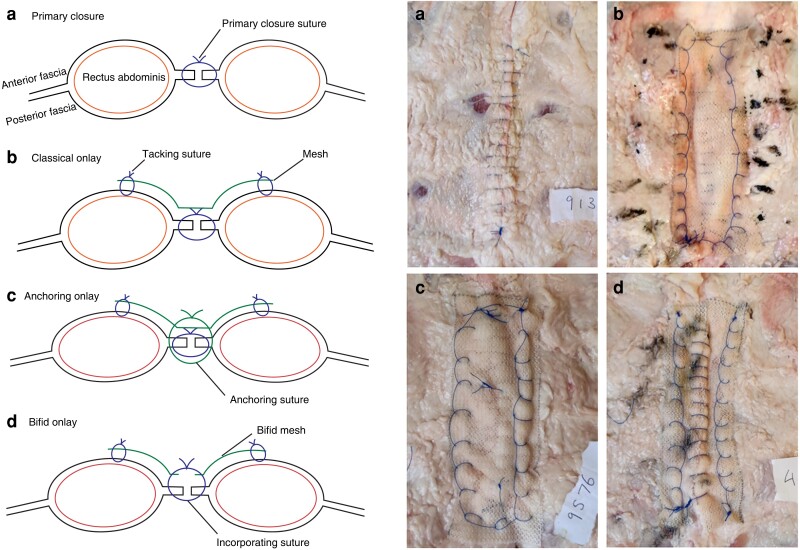
Abdominal wall closure types **a** Primary closure of linea alba with running small-bite (5 mm separation, 5 mm width), continuous 2/0 Prolene^®^. **b** Classical onlay with 6 × 15-cm TIGR® mesh. The midline is closed primarily, and the mesh is then fixed in place over the closure with a continuous 2/0 Prolene^®^ suture. **c** Anchoring onlay brings the addition of three, full-thickness interrupted 2/0 Prolene^®^ sutures that anchor the mesh in the midline. **d** Bifid onlay begins by tacking two 3 × 15-cm mesh leaflets in place, one either side of the midline. The linea alba is then closed, incorporating the mesh on both sides as a buttress.

For the bifid technique, the mesh was cut evenly along its length, leaving two 15 × 3-cm strips. A strip of mesh was tacked to the anterior fascia on either side of the incision using a running, locking suture such that the cut edge overlapped the abdominal incision. A small-bite, primary closure was then performed, taking linea alba, and mesh on either side of the incision, effectively incorporating the two strips of mesh into the closure for a buttress effect (*Fig. 1*).

A single, general surgery specialist registrar performed all closures. Technical and clinical training in all prophylactic mesh placement techniques used here had been provided by consultant surgeons. For each closure, the time for completion was recorded. The Kruskal–Wallis test in GraphPad Prism 9.4.1™ (GraphPad Software, San Diego, California, USA)^®^ was used to analyse the statistical significance (2-sided 5 per cent) of differences in preparation times between groups. All samples were tested on the biomechanical abdominal wall model and the modified ball burst test immediately after preparation. Primary outcomes were time taken for closure completion (minutes, seconds), transverse stretch (Δ), and burst force (Newtons).

### Biomechanical abdominal wall model

The surrogate abdominal wall model has been described previously^16,17^. A porcine abdominal wall was stretched over a box-shaped rig. This represented the abdominal wall and cavity. An oversized balloon contained within the box was inflated with a controlled compressed air supply to standardized pressures, designed to represent physiological intra-abdominal pressures (IAPs). Pressures within the rig were increased in 2-kPa (15-mmHg) increments to a maximum of 24 kPa (180 mmHg). Each increment was held for 10 s, ensuring quasistatic conditions.

**Fig. 2:**
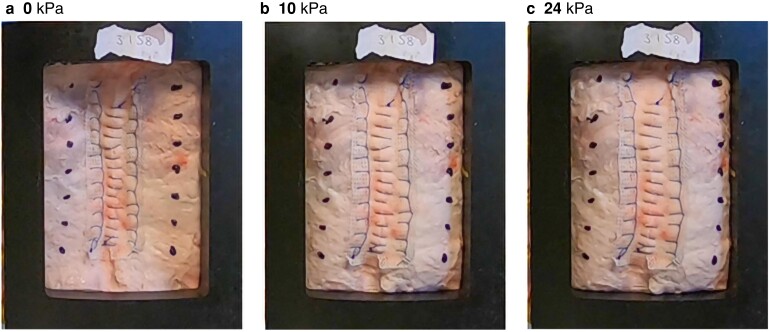
Biomechanical abdominal wall model Example of video capture stills used for transverse stretch analysis. The sample is mounted in a box rig, which stretches the abdominal wall across an inflated, oversized balloon at set pressure intervals. Intrapair distance is calculated for dot-pairs across pressure values and compared with the distance at 0 kPa. Bifid mesh samples at **a** 0 kPa, **b** 10 kPa, and **c** 24 kPa.

Transverse stretch was measured using dot-tracking video analysis (GoPro Hero^®^ 7 and 8 cameras - GoPro, San Mateo, California, USA) of the anterior abdominal wall captured during testing. Dot-pairs were painted on to the abdominal wall with black acrylic paint (*Fig. 2*). These were placed 4 cm lateral to the incision, at 2-cm intervals, beginning at the top of the mesh. Still images were extracted from the video footage at the end of each 10-s interval. A custom MATLAB^®^ (Mathworks, Natick, Massachusetts, USA) script was used to measure transverse stretch by comparing dot-pair distances at 0 kPa with those at each pressure increment. Transverse stretch was expressed in terms of dot-pair distance at a given pressure, divided by original length at 0 kPa. Mean stretch for each pressure value was calculated in each group by averaging values across all dot-pairs for that closure method, allowing comparison between groups as opposed to between individual samples. Outcomes were summarized numerically and graphically.

### Modified ball burst test

A custom ball burst rig was developed using a three-dimensionally printed rectangular polylactic acid mounting frame with a metallic mounting vice and a 7-cm diameter steel ball loaded on a Zwick Roell ProLine Twin Column (Zwick Roell, Ulm, Germany) testing machine. Samples were mounted such that the ball applied its load through the posterior abdominal wall. The ball head was placed with its centre over the middle of the incision. The ball advanced at a rate of 5 mm/min until a preload of 0.1 N was reached. From this point, a standard rate of travel of 10 mm/min was set to provide quasistatic loading, with the ball head applying load to the sample, until a 20 per cent drop-off in force compared with the maximum recorded was observed. The maximum force observed denoted the burst force. Data for time, travel distance, and force were collected at 0.6–0.7-s intervals throughout each test. Observations regarding tissue tearing, and suture and mesh failure were recorded for each sample.

## Results

### Sample randomization and preparation

A total of 18 samples were assigned randomly to the test groups. Four each were assigned to the primary closure and classical onlay groups, and five each to the anchoring and bifid groups. Early testing required defrosting periods longer than 24 h owing to tissue bulk and inconsistent thawing. This resulted in three samples—two from the anchoring and one from the bifid groups—having early evidence of putrefaction.

**Fig. 3:**
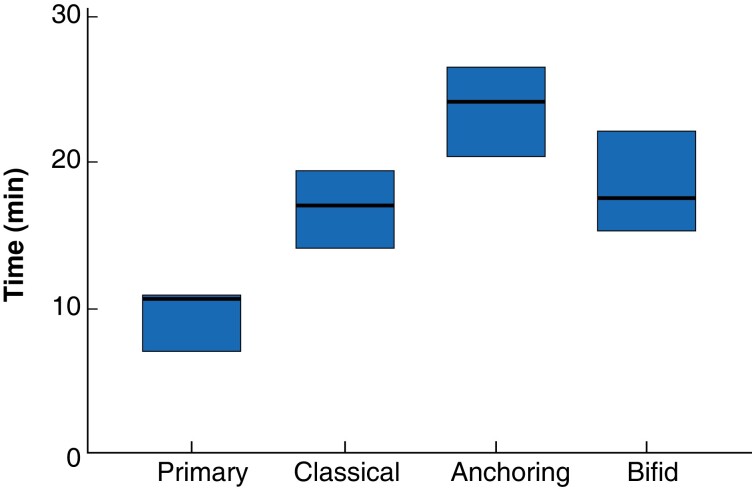
Box plot showing time taken to perform each closure technique Bold lines show median value, and boxes indicate range.

Preparation times varied between groups. Primary closure was fastest (mean 9 min 51 s (s.d. 1 min, 54 s)) and anchoring the slowest (24 min, 1 s (2 min, 21 s)). Classical and bifid closures took similar times to complete, taking a mean of 16 min, 54 s and 18 min, 7 s respectively, with significant overlap between sample preparation times (*Fig. 3*). The Kruskal–Wallis test confirmed a difference in completion times between closure methods (*P* < 0.001), without indicating which was stochastically dominant. Simple comparison of means suggested this was the primary closure group.

### Biomechanical abdominal wall model

**Fig. 4:**
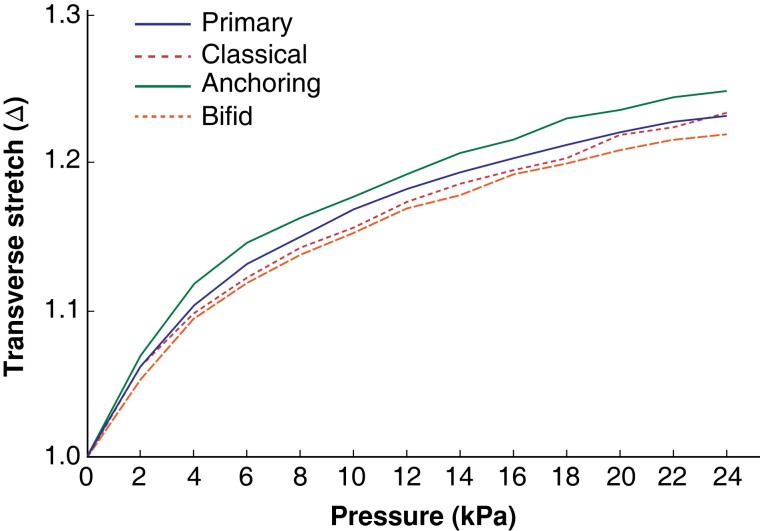
Abdominal wall model transverse stretch series: overall comparison between groups of transverse stretch across 2-kPa pressure intervals (0–24 kPa) Mean intrapair distance was calculated for each pressure interval using all dot-pairs within a treatment arm.

All 18 samples were tested on the abdominal wall model immediately after the closure had been completed. For comparison between groups, the mean transverse stretch was calculated using all dot-pairs across samples in a given group for all pressure values (26 dot-pairs for primary, 28 for classical, 32 for anchoring, and 32 for bifid groups). The total number of dot-pairs across all groups was 118. At 24 kPa, mean(s.d.) transverse stretch (Δ) was 1.231(0.034) for primary, 1.232(0.035) for classical, 1.245(0.034) for anchoring, and 1.226(0.066) for bifid closure (*Fig. 4*).

### Modified ball burst test

After testing on the abdominal wall model, each sample was mounted on the modified ball burst platform. The mean(s.d.) bursting force was similar for primary closure (702(87) N), anchoring onlay (706(145) N), and bifid onlay (710(245) N) groups, but there was significant variation within each group. The classical onlay technique (853(152) N) performed best on average. The best performing and worst performing samples were in the bifid group; these failed at 1053 and 493 N.

**Fig. 5:**
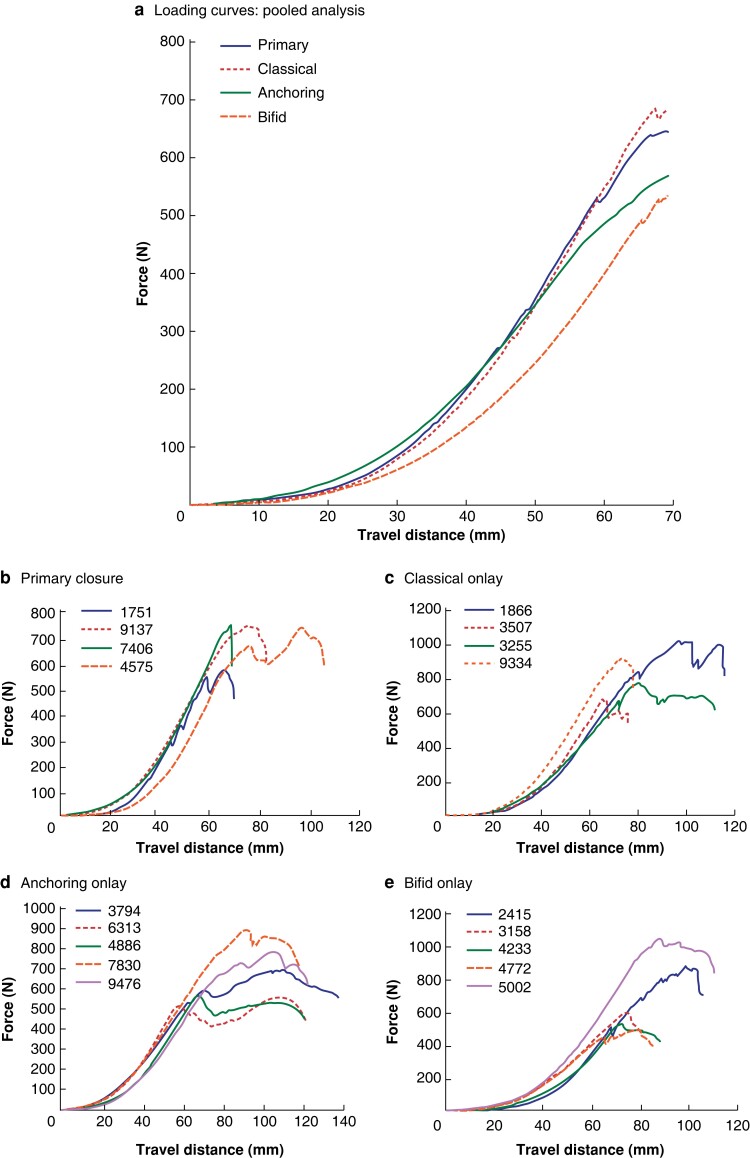
Modified ball burst loading curves **a** Pooled analysis of modified ball burst test results. Travel distance was limited to 70 mm for comparison between treatment arms to assess loading characteristics before failure points. **b–e** Subgroup analysis for each closure method demonstrating failure patterns: **b** primary closure, **c** classical onlay, **d** anchoring onlay, and **e** bifid onlay. The numbers included in the legends for **b**–**e** are sample identifiers created during randomisation.

Loading patterns were interrogated during the modified ball burst test by comparing force and travel distance. As there was significant variation in burst force and travel distance across samples, a cut-off of 70 mm travel distance was used for intergroup analysis. No samples failed before this travel distance, allowing comparison of initial loading characteristics before failure effects. The bifid technique demonstrated a more compliant loading curve, with lower forces recorded at equivalent travel distances compared with other methods (*Fig. 5*). Primary closure tended towards an abrupt failure point, demonstrated by a sharp drop-off in force on the load curves. By comparison, all three mesh techniques showed a trend towards more gradual failure, often without a sudden failure point.

**Fig. 6:**
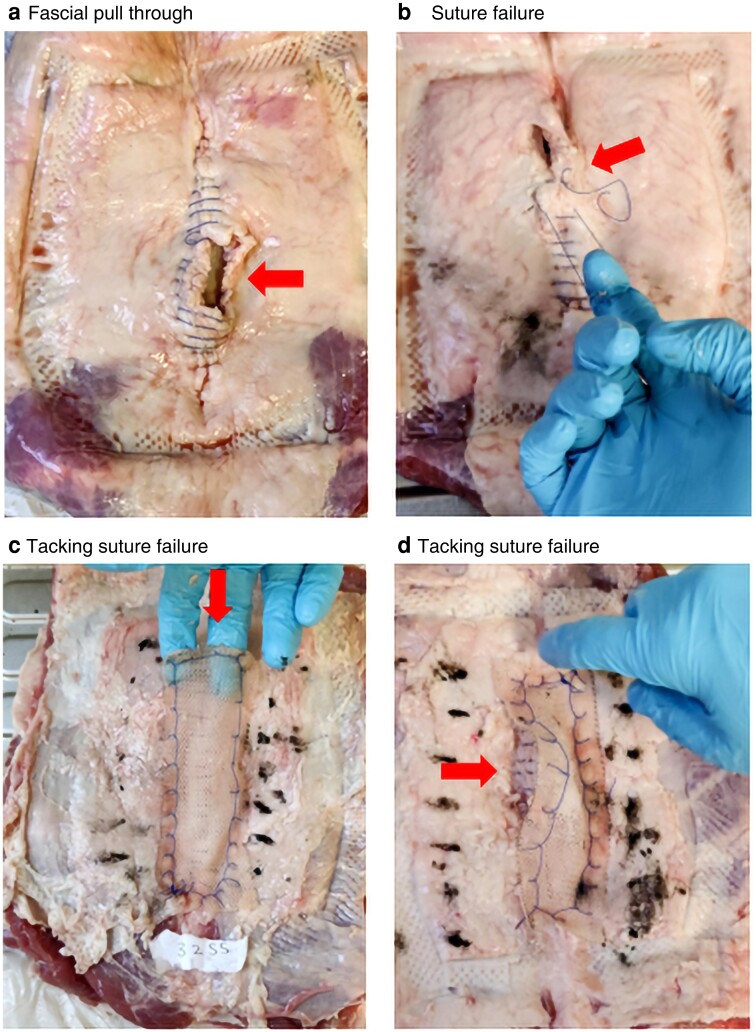
Examples of failure patterns **a** Suture material pulled through fascial tissue, **b** suture failure, and **c**,**d** circumferential tacking suture pull-through.

Inspection of the samples after testing demonstrated multiple modes of technical failure, including suture or knot failure at the primary closure, tacking suture pull-through, and fascial pull-through (*Fig. 6*). Most samples failed when the surrounding tissues gave away, as opposed to failure of the closure.

## Discussion

The results of this study showed that each approach to mesh augmented closure can be performed with minimal additional time compared with that for primary closure. All closure types demonstrated similar transverse stretch properties when placed on the abdominal wall model, with no clearly superior approach established. Performance on the modified ball burst model was again comparable, though the classical approach had the highest mean burst force (mean(s.d.) 853(152) N). The bifid approach showed the greatest variability across the samples (710(245) N), as well as a more compliant loading curve. Failure patterns closely related to burst forces, with sudden failures typically being a consequence of primary suture-line failure or fascial pull-through.

Under normal anatomical conditions, average resting IAP in healthy adults is 1.6 mmHg^18^. With coughing, Valsalva manoeuvre, and jumping, it can reach 107, 64.9, and 171 mmHg (equivalent to 22.8 kPa) respectively^18^. Perioperative IAP rises to a peak of 1.74 kPa on a patient wakening from anaesthesia^17^. The biomechanical abdominal wall model used here sequentially increased pressures on the closures to a maximum of 24 kPa, far exceeding physiological IAP values. Despite this, no closure failures were seen at this stage of testing across any samples.

The minimum burst force seen during testing was 493 N. This force was delivered directly on to the closure and concentrated through the spherical indenter. In layperson’s terms, this is equivalent to a 50-kg person balancing their weight directly on to the wound through only their fist. The best performing repair, at 1053 N, would have held up to a 100-kg weight. The stress–strain relationship, which describes overall tissue elasticity (Young’s modulus), is challenging to define for soft tissue, and even more so for complex combined biological and synthetic constructs, such those created by the abdominal wall–mesh interface. The success or otherwise of a given closure method is as dependent on local tissue failure properties as it is on the elasticity of the construct. The ball burst test provides a simple, robust approach by which to compare these complex constructs in a meaningful way.

Even considering the limitations of this study, notably the small sample sizes and initial issues around sample thawing, the findings demonstrated the ability of the repairs to stand up to supraphysiological stresses.

Both of these biomechanical models test the closures under quasistatic conditions, with gradual sustained increases in the forces involved. In many circumstances, such as coughing, jumping, and laughing, the abdominal wall does not experience this kind of sustained, gradually increasing load, but rather undergoes rapid, repetitive increases and decreases. Dynamic, repeated loading simulating coughing produces distinct failure patterns in rat incisional hernia models. These failures are influenced by peak load and duration^19^. The influence of dynamic loading should be tested in future.

The failure patterns seen in the test benches fall into two major categories—suture failure or tissue failure. Tissue failure was likely a consequence of the forces fatiguing the organic tissues, whereas suture failures were either a direct result of a suture or knot breaking or cutting through the tissue into the midline wound. A similar phenomenon has been demonstrated in animal models comparing mesh and primary closure, with failures in the mesh group occurring at the wound healing interface^20^. Further animal models and clinical studies have demonstrated that early fascial gaping can predict incisional hernias long before they manifest clinically. This gaping, identifiable either by CT or the serial X-ray monitoring of migration of metallic clips placed on fascial edges, is likely the consequence of early clinically occult fascial dehiscence^21,22^.

The presence of a mesh across the incision may act as a second line of defence in the event of primary suture failure or fascial dehiscence. The bifid group showed the most intragroup variability and a more compliant loading pattern. These properties may be attributable to a unique buttress effect provided to the suture line, like that sought by use of pledgets in vascular surgery. However, as it lacks the second line of defence in the event of a technical suture failure, such as a snapped knot, it cannot compensate like the other mesh approaches.

This study did not investigate mesh positioning, but rather looked solely at the onlay approach. The onlay approach is simpler to perform than the sublay and retrorectus approaches. The PRIMA RCT^12^ compared onlay and sublay approaches. Onlay outperformed the sublay approach with regards to incisional hernia formation at 2 years (13 *versus* 18 per cent respectively), although seromas were more frequent. Both approaches were superior to primary closure (30 per cent). The recently published long-term results of the PRIMAAT RCT^23^ demonstrated a 0 per cent cumulative incidence of incisional hernia at 60 months with use of prophylactic retrorectus mesh placement at laparotomy closure for abdominal aortic aneurysm repair, compared with 49.2 per cent with primary closure alone.

The STITCH^24^ and PRIMA^12^ trials have shown that technical differences can result in significant variation in clinical outcomes. However, the development of incisional hernias comprises a complex interplay between patient, technical, and disease-related factors. Early occult or clinically apparent fascial dehiscence is likely a consequence of either technical failure—either of the material used or the quality of knots—or tissue failure resulting from poor tissue quality. Attention to basic surgical principles, namely sound knot-tying, atraumatic tissue handling, appropriate tissue apposition, and use of a laparotomy wound bundle, has a significant role to play in the prevention of these early wound failures^25^, and should provide the basis on which more complex adjuncts such as mesh augmentation are built.

The approaches to prophylactic mesh placement interrogated here have comparable biomechanical properties. The placement of mesh takes up to 15 min longer than primary closure. Suture failure patterns demonstrate the merits of each approach, with the bifid onlay method reinforcing poor-quality surrounding tissue, and the classical and anchoring onlay techniques providing a second line of defence in the event of failure of the primary closure. This may suggest different optimum uses for each approach. The supraphysiological forces and pressures used in this study demonstrate the mesh’s ability to withstand extreme quasistatic loads.

## Data Availability

Data access may be requested by writing to the lead author. The full study protocol is available on request from the corresponding author.
